# Cellular Reprogramming Using Protein and Cell-Penetrating Peptides

**DOI:** 10.3390/ijms18030552

**Published:** 2017-03-03

**Authors:** Bong Jong Seo, Yean Ju Hong, Jeong Tae Do

**Affiliations:** Department of Animal Biotechnology, College of Animal Bioscience and Technology, Konkuk University, Seoul 143-701, Korea; sbj1990@naver.com (B.J.S.); ndhong7@gmail.com (Y.J.H.)

**Keywords:** cell penetrating peptide, reprogramming, protein, iPSCs

## Abstract

Recently, stem cells have been suggested as invaluable tools for cell therapy because of their self-renewal and multilineage differentiation potential. Thus, scientists have developed a variety of methods to generate pluripotent stem cells, from nuclear transfer technology to direct reprogramming using defined factors, or induced pluripotent stem cells (iPSCs). Considering the ethical issues and efficiency, iPSCs are thought to be one of the most promising stem cells for cell therapy. Induced pluripotent stem cells can be generated by transduction with a virus, plasmid, RNA, or protein. Herein, we provide an overview of the current technology for iPSC generation and describe protein-based transduction technology in detail.

## 1. Introduction

Stem cells are able to maintain their stemness and proliferate as required to replace dead cells in the body. Among stem cells, pluripotent stem cells are potent cells that can give rise to any cell type in an organism. Pluripotent stem cells can be derived not only from early embryos, such as blastocysts and epiblasts, but also from differentiated cells via reprogramming [1]. Before Yamanaka discovered the reprogramming factors that induce pluripotency [1], reprogramming studies focused on reprogramming by somatic cell nuclear transfer (SCNT). However, SCNT suffered from several shortcomings, such as its technical difficulty, ethical problems, and low efficiency. Thus, egg-free reprogramming approaches have been developed, such as reprogramming by cell fusion in embryonic stem cell extract [2,3], and transduction of defined factors [4]. In the quest for methods to induce pluripotency, several studies revealed that reprogramming factors reside in the nucleus. First, only enucleated metaphase II (MII) oocytes (lacking a nuclear envelope), but not enucleated zygotes (with a nuclear envelope) were able to reprogram somatic cells successfully after nuclear transfer [5,6]. Second, karyoplasts, but not cytoplasts, of embryonic stem (ES) cells were able to reprogram somatic cells after cell fusion [7]. These sequential experiments suggested that nuclear factors are responsible for cellular reprogramming, which was finally proven by the generation of induced pluripotent stem cells (iPSCs) using transcription factors [4]. Since then, the identified reprogramming proteins could be used to reprogram cells directly after being introduced into somatic cells.

## 2. History of Exogene-Free Induced Pluripotent Stem Cells

**Figure 1:**
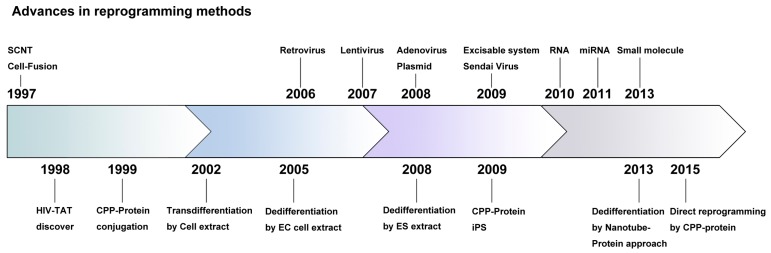
Timeline of reprogramming research. The upper panel represents advances in non-protein-based reprogramming approaches, while the bottom panel represents protein-based reprogramming approaches.

Studies on reprogramming began with the development of nuclear transfer technology and, subsequently, factors were identified that could induce reprogramming without oocytes [4]. As a result, iPSC generation using retroviral transduction was developed; however, it presented some challenges, the most important of which was the integration of exogenous transgenes into the host genome after viral transduction [8]. Ultimately, iPSC research aims to develop regenerative and therapeutic applications to cure intractable human diseases; therefore, this issue must be resolved before clinical trials. To minimize transgene integration, diverse alternative reprogramming methods have been developed (Figure 1). Yamanaka et al. used plasmids instead of viruses to transduce reprogramming factors to overcome the integration problem [8]. Two separate plasmids, containing Oct4 (Octamer-binding transcription factor 4)-Sox2 (Sex determining region Y-box 2)-Klf4 (Kruppel-like factor 4) and c-Myc (Myc proto-oncogene, bHLH transcription factor), respectively, were introduced into somatic cells using a transfection reagent [8]. Stadtfeld et al. also succeeded in generating iPSCs lacking exogenous integration using an adenoviral vector as the carrier for the reprogramming factors (Oct4, Sox2, c-Myc, and Klf4) [9]. In a preclinical study, Soldner et al. generated integration-free iPSCs from fibroblasts of a Parkinson’s disease patient using the Cre-recombinase excisable system. These patient-derived iPSCs maintained their pluripotency successfully after the excision of exogenous sequences, which were introduced by a viral infection of the reprogramming genes [10]. A single polycistronic vector containing the c-Myc-Klf4-Oct4-Sox2 (MKOS) reprogramming cassette flanked by loxP was generated in both humans and mice [11]. The exogenous MKOS could be removed by Cre-recombinase treatment [11]. In addition, the transposon-based piggyBAC transposition system was also used for integration-free iPSC generation in mice and humans [12]. 

Although the exogenous reprogramming factors are removed after reprogramming, residual exogenous sequences originating from the virus still remain in the host genome [13]. For this reason, some new reprogramming approaches were attempted that did not use DNA vectors. Using Sendai virus (SeV), a negative-sense single-stranded RNA vector could be used for reprogramming, without the risk of exogene integration [14]. Sendai virus enabled the reprogramming of human fibroblasts [15] or T-cells [16] more efficiently than retrovirus- or lentivirus-mediated technologies. Some groups used episomal plasmid vectors to generate human iPSCs, with no integration of vector or transgene sequences [17,18].

By contrast, RNA-based reprogramming has been developed as an alternative plan that avoids antiviral responses and integration problems [19,20,21]. MicroRNA (miR) 302/367 clusters, which are expressed highly in pluripotent stem cells, were used to reprogram mouse and human fibroblasts [22]. Recently, it was reported that iPSCs could be induced by combinations of small molecules. However, this chemical reprogramming approach needs to be standardized and some aspects require further development, such as the exact mechanism of small molecule-mediated reprogramming, the side effects of the chemicals, and their low efficiencies.

## 3. Protein-Based Reprogramming Approaches

An alternative way to overcome the above-mentioned disadvantages is the use of proteins for cellular reprogramming (Table 1). The probability that proteins could induce reprogramming was suggested more than a decade ago. Cell extracts are believed to comprise the nuclear regulatory components needed to induce nuclear reprogramming and, thus, drive cell fate transition. Some researchers have demonstrated the cell fate transition ability of protein extracts. Somatic cells could transdifferentiate into another somatic cell type by incubation with extracts of primary or transformed human T-cells, or neuronal precursors. These transdifferentiated cells exhibited characteristics of the target cells instead of their original cell type [23,24,25]. 

Somatic cells can also be reprogrammed into a pluripotent state after treatment with an extract of human NCCIT embryonic carcinoma (EC) cells. The reprogrammed cells expressed Oct4, a major pluripotency marker, and could differentiate into multi-lineage cell types, proving their acquisition of pluripotency [26]. They also showed demethylation of the *Oct4* and *Nanog* (Nanog homeobox) regulatory region and overall histone modification, indicating that epigenetic states could also be reprogrammed to the pluripotent state using a cell extract-mediated approach [27]. The following year, a reprogramming approach using extracts of embryonic stem cells (ESCs) were able to activate pluripotency genes, *Oct4*, *Sox2*, *Klf4*, *c-Myc*, and *Nanog* in 293T cells [3]. The reprogrammed cells acquired the ability to self-renew and showed the developmental potential of all three germ layers. In 2010, researchers used ESC protein extracts to reprogram adult cardiac fibroblasts. These protein-iPSCs showed typical pluripotency features, including gene expression and epigenetic patterns, as well as in vivo and in vitro differentiation potentials. In particular, they revealed that protein-iPSCs could undergo full-term development through tetraploid complementation, the most stringent assay for proving pluripotency. Another noteworthy point of this research was that the single transfer of ESC-derived extract protein was sufficient to induce pluripotency in adult, but not fetal, somatic cells [28].

**Figure 2:**
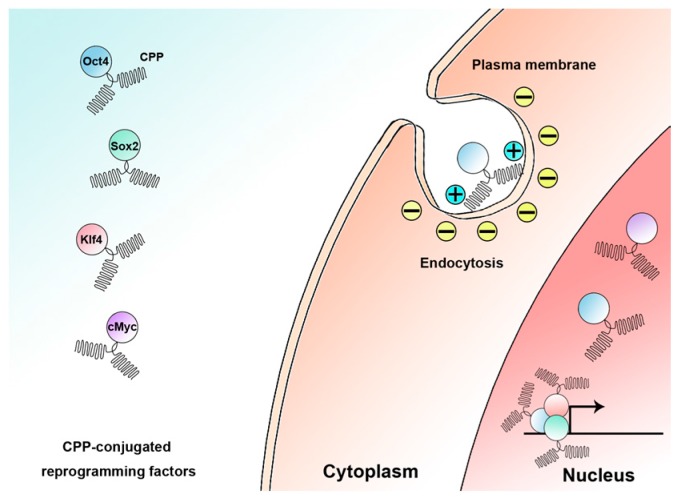
Cellular uptake mechanism of cell-penetrating peptides (CPP)-conjugated proteins. The positively-charged amino acid residues of the CPP interacts with the negatively-charged cell membrane constituents and enables the target protein to be taken up into cytosol via endocytosis. c-Myc: Myc proto-oncogene, bHLH transcription factor; Klf4: Kruppel-like factor 4; Oct4: Octamer-binding transcription factor 4; Sox2: Sex determining region Y-box 2.

However, the main problem with this approach is the delivery of proteins into the intracellular space, because of the large size of proteins and the hydrophobic property of the cellular membrane. Macromolecules, such as proteins, penetrate the plasma membrane poorly. Therefore, somatic cells have to be pretreated with cell permeabilization agents for reversible permeabilization, which transiently makes holes in the cell membrane to allow the proteins to pass. This procedure is very harmful in terms of cell survival and, thus, affects the efficiency of reprogramming. In 1988, Flankel and Pabo discovered that the purified human immunodeficiency virus trans-activator of transcription (HIV-TAT) protein could flow into cells [29]. Other peptides, such as VP22 and penetratin, have also been reported to penetrate the cell membranes [30,31]. These peptides were termed cell-penetrating peptides (CPPs) because of this distinct property. Based on their physicochemical properties, CPPs can be classified into three types: amphipathic, hydrophobic, and cationic. Based on their origin, CPPs can also be categorized into three types: designed peptides, natural protein-derived peptides, and chimeric peptides. They are also known as protein transduction domains (PTDs). One class of the CPP are enriched in basic amino acids, lysines, or arginines, which are positively charged, allowing them to interact with negatively-charged phospholipids in the cell membrane (Figure 2). Currently, researchers are investigating methods to deliver proteins into the intracellular space by fusing them with CPPs.

## 4. Development of Protein Transduction Technology

In 1999, Schwarze et al. fused the 11-amino acid HIV-TAT (GRKKRRQRRRPQ) protein transduction domain with a biologically active β-galactosidase protein as well as a fluorescein isothiocyanate (FITC)-Gly-Gly-Gly-Gly motif, resulting in a 120-kDa fusion protein (TAT-β-gal) and 15-oligomer peptide (TAT-FITC), respectively. Both, TAT-β-gal and TAT-FITC successfully transduced into the cultured cells. Moreover, they showed the in vivo transduction ability of fusion proteins via intraperitoneal injection into mice and found that these proteins could be successfully delivered into all tissues [32]. 

Applying this methodology, a number of researchers have synthesized a diverse version of CPP fusion proteins, including transcription factors. For example, recombinant TAT-HOXB4 (Homeobox B4) protein enables rapid ex vivo expansion of hematopoietic stem cells (HSCs), which was comparable to HOXB4 retrovirus [33]. Moreover, these TAT-HOXB4-expanded HSCs retained multilineage differentiation potential. The endodermal development-related factor PDX1 conjugated with TAT (TAT-PDX1) could be transferred into human embryonic stem cells (hESCs) followed by activation of the target insulin gene [34]. A cell-permeant form of Nkx2.2 proteins was used to increase oligodendroglial differentiation of mouse ESC-derived neural stem cells (NSCs). This fusion protein was composed of the Nkx2.2 (NK2 Homeobox 2), a nuclear localization signal (NLS), and the TAT domain [35]. The efficiency of oligodendrocyte differentiation was comparable to that observed in lentiviral transduction.

With regard to pluripotency factors, Manal et al. generated cell-permeant Oct4 and Sox2 proteins by fusing them with TAT peptide. Transducible Oct4 and Sox2 proteins could bind their DNA target sequence and thus regulate transcription. Interestingly, the knockdown effect of *Oct4* or *Sox2* by short interfering RNA (siRNA) treatment in mouse ESCs could be compensated by culturing with Oct4 and Sox2 fusion proteins. This study suggested the possibility for CPP-conjugated-reprogramming factor protein transduction into cells without genetic integration [36]. 

## 5. Reprogramming via Cell-Penetrating Peptide-Mediated Protein Transduction

Before CPPs were used as strong tools for reprogramming, many research groups focused their efforts on establishing efficient CPP-mediated protein delivery systems and their related mechanisms. To date, over 100 different kinds of CPPs have been reported by numerous laboratories. Cell-penetrating peptides can be categorized by their physicochemical characteristics or their origins. In the field of cellular reprogramming, natural protein-derived or synthetic cationic peptides are used commonly, such as the transactivator of transcription (TAT, derived from the human immunodeficiency virus) or C-terminal fused undeca-arginine (11R) (Table 1).

### 5.1. Groundbreaking Success in Reprogramming by Using Cell-Penetrating Peptides

In 2009, the first successful attempt in CPP-based reprogramming to induce mouse pluripotent stem cells was reported by Zhou et al. [37]. They used 11R fused reprogramming factors (11R-RFs) for virus-free reprogramming, with or without valproic acid (VPA), which is a histone deacetylase (HDAC) inhibitor. An Oct4-GFP (green fluorescent protein) reporter in mouse embryonic fibroblast (MEF) cells was activated and pluripotency-related genes were expressed, which was accompanied by epigenetic changes. Differentiation to the three germ layer stage in vitro and contribution to various tissues in chimeras confirmed the successful induction of pluripotency by protein-based reprogramming. 

**Figure 3:**
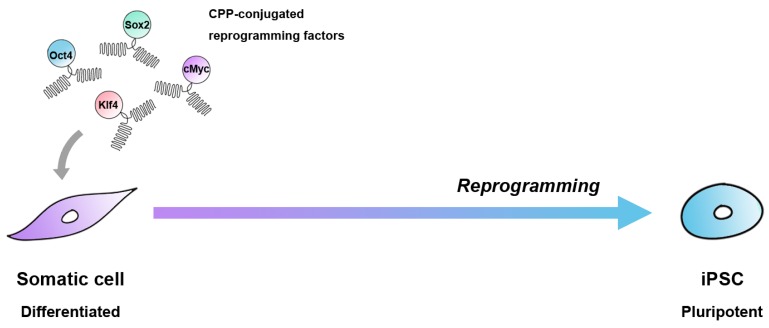
Reprogramming of somatic cells using CPP-conjugated proteins. Transduction of reprogramming proteins with a CPP may induce pluripotency in somatic cells. iPSC: Induced pluripotent stem cell.

Experiments in human cells using a similar approach were conducted by Kim et al. in the same year [38]. For reprogramming, they established stable HEK293 cell lines expressing Oct4, Sox2, Klf4, and c-Myc fused with nona-arginines (HEK293-4F-9R) (Figure 3). After three to four rounds of treatment (16 h treatment followed by a six-day incubation in ESC culture medium) of human newborn fibroblasts with the HEK293-4F-9R cell extract, they obtained iPSC-like cells. Although the reprogramming efficiencies reported by both Zhou et al. [37] and Kim et al. [38] (approximately 0.001%) were lower than virus-based (0.01%) reprogramming, the established protein-reprogrammed human induced pluripotent stem cells (p-hiPSCs) displayed similar characteristics to human ESCs in terms of morphology, proliferation, and expression of pluripotency markers. Established p-hiPSCs were maintained for more than 35 passages and showed in vitro and in vivo differentiation potential to all three germ layers. Furthermore, these p-hiPSCs could differentiate specifically into functional dopaminergic neurons, which could rescue the motor deficits in a Parkinson’s disease rat model [39].

### 5.2. Efforts to Increase Cell-Penetrating Efficiency Using the Other Peptides

In 2012, Lee at al. found differences of the specific gene expression patterns in iPSCs induced by viral versus CPP-based delivery of the reprogramming factors. Initially, they found that an irrelevant retroviral vector could accelerate CPP-based delivery efficiency. In a previous report, McWhirter et al. suggested that toll-like receptors (TLRs) could be activated by viral infection [40]. They found that activation of TLR3 signaling by retroviral infection could enhance the efficiency of CPP-based reprogramming [41].

Another common CPP, TAT, was used for reprogramming by Thier and colleagues [42,43]. They established conditions for the generation of cell-permeant recombinant fusion proteins and their delivery into somatic cells; the Oct4 or Sox2 proteins were fused with TAT peptide. The Oct4-TAT protein could substitute for Oct4-encoding virus during the generation of iPSCs. At the initial trial, the authors infected MEF cells with viruses encoding Sox2, Klf4, and c-Myc, followed by incubation with Oct4-TAT for 14 days, which resulted in Oct4-GFP-positive colonies at day 16 post‑treatment [42]. Thereafter, they constructed a Sox2-TAT recombinant protein that could also substitute for Sox2-expressing virus [43]. Based on these reports, they claimed that recombinant proteins fused with TAT could substitute for viral vectors in the reprogramming process. 

Zhang et al. tried another approach for reprogramming using five RFs (Oct4, Sox2, c-Myc, Klf4, and Nanog) fused with 11R and TAT [44]. Consequently, they obtained iPSC-like colonies almost two weeks after transduction of CPPs from human foreskin fibroblasts. Intriguingly, using 5-TAT-RFs in conjunction with the epigenetic modifier VPA, the efficiency of generation of iPSC-like colonies increased to approximately 0.012%, which was significantly higher than that of previous reports until 2012 [37,38].

### 5.3. Cell-Penetrating Peptide-Based Direct Lineage Conversion

Direct lineage conversion is the process of transdifferentiation of a differentiated cell type to another differentiated cell type. As with the pluripotent reprogramming by CPP-based proteins, direct lineage conversion could also be induced by CPP-based proteins. Islas et al. tried to reprogram human dermal fibroblasts (HDFs) into cardiac progenitors using TAT-ETS2 (erythroblastosis virus E26 oncogene homolog 2) and TAT-MESP1 (mesoderm posterior 1) fusion proteins [45]. Dai et al. used recombinant TAT-transcription factors, such as TAT-Oct4, TAT-Klf4, and TAT-Sox2, together with small molecules, RG108 (DNA methyltransferase 1 inhibitor) and purmorphamine (smoothened inhibitor), for the direct conversion of human adipose-derived stem cells (ADSCs) into corneal endothelia (CE)-like cells [46]. Recently, Hu et al. discovered that C-end rule (CendR), a cell-penetrating peptide, which coupled with Sox2, could be used to reprogram pigmented epithelial (RPE) cells into functional neurons [47]. Li et al. reported that QQ-reagent (a protein transduction reagent)-modified cardiac transcription factors, such as mouse Gata4 (GATA binding protein 4), Hand2 (heart- and neural crest derivatives-expressed protein 2), Mef2c (myocyte-specific enhancer factor 2C), and Tbx5 (T-box transcription factor 5) (mGHMT), were sufficient to reprogram HDFs into cardiac progenitor cells (CPCs), which could improve cardiac function after myocardial infarction [48].

### 5.4. Various Approaches for Protein Transduction in Reprogramming

There have been several attempts at protein-based reprogramming using non-CPP-based methods for protein delivery. Our laboratory was the first to test a nanocarrier to deliver proteins into somatic cells for cellular reprogramming [49]. We found that titanium oxide (TiO_2_) nanotubes could be used to deliver reprogramming factors directly into fibroblasts and showed neither cytotoxic effects nor harmful effects on cell proliferation. Although iPSC lines could not be established using this nanotube–protein conjugation system, delivered reprogramming factors could activate the pluripotency biomarker, Oct4-GFP, and induce the formation of ESC-like colonies [49]. In another case, Lim et al. reported the use of macromolecule intracellular transduction technology (MITT) for protein delivery and reprogramming [50]. They fused various hydrophobic macromolecule transduction domains (MTDs), such as MTD47, MTD52, MTD84, MTD86, MTD132, MTD173, and MTD181, with RFs, such as Oct4, Sox2, Klf4, c-Myc, Nanog, and Lin28 (lineage protein 28) (with Vitamin C) to reprogram HDFs. Although the HDFs were not fully reprogrammed, the efficiency of partial reprogramming using this protocol was about 0.074% (with 1 µg/µL vitamin C) and 0.34% (with 10 µg/µL vitamin C).

## 6. Limitations of Cell-Penetrating Peptide-Mediated Reprogramming

Cell-penetrating peptide-based reprogramming might be a safe way to induce reprogramming; however, its low efficiency compared with other methods is a significant concern. The main problem is the poor stability of the recombinant proteins and following endocytic uptake. These points are critical to increasing the efficiency of reprogramming using CPP-based protein transduction. Thier et al. focused on medium optimization for the delivery of a cell-permeant Oct4 protein [42]. They suggested that the combination of fetal calf serum and serum replacement in KnockOut D-MEM (Invitrogen) media increased the stability of Oct4-TAT fusion protein significantly.

Innovative nanocarriers, such as gold nanoparticles, in conjunction with CPP, could enhance intracellular translocation [51]. Using these kinds of approaches, the strength of CPP-based reprogramming could be maximized.

Although it is clear that CPPs promote the cellular uptake of various proteins in the simple culture system, the detailed mechanism of CPP internalization remains to be determined. To date, although the exact mechanisms remain unsolved, the endocytosis pathway is thought to be the major CPP internalization mechanism [52]. Another issue that should be resolved is the unknown subsequent side effects of CPPs on the treated cells and neighboring cells after transplantation of the treated cells. This issue is very important for the clinical application of cells generated by CPP-based technology.

## 7. Conclusions

The field of stem cells and regenerative medicine is undergoing rapid technological development. In particular, cellular reprogramming has been one of the fastest progressing areas in recent years. Since the discovery that CPPs internalize into cells, there have been many efforts to apply this technology. More efficient versions of CPPs have been synthesized and used for cellular reprogramming. By contrast, recently, some researchers have attempted to find novel peptides that are cell-type specific: a specific CPP would be used to in its matched somatic cell type for protein transduction. Although technical problems remain, we are hopeful that protein-based exogene-free iPSCs could be used for future tissue replacement therapy. 

## Figures and Tables

**Table 1:** Summary of non-integrative methodologies using proteins.

Source of Somatic Cells	Reprogramming Factors	Type of Protocol	Assessment of Pluripotency	Efficiency	Reference
293T or NIH3T3 fibroblasts	Extract of human NCCIT ECCs or mouse ESCs	Exposed to cell extract	Pluripotency		[26]
293T	Extract of mouse ESCs				[3]
Mouse cFBs	Extract of mouse ESCs				[28]
MEFs	OKSM with VPA	11R-fused CPPs		0.001%	[37]
HNFs	OKSM	9R-fused CPPs		0.001%	[38]
Human fibroblast	OS with PolyI:C	11R-fused CPPs			[41]
MEFs	Oct4 with Serum replacement, Sucrose	TAT-fused CPPs			[42]
MEFs	Sox2 with Serum replacement	TAT-fused CPPs			[43]
HAF cells HFFs NIH3T3 fibroblasts	OKSMN with VPA	TAT-fused CPPs		0.012%	[44]
HAF cells HFFs NIH3T3 fibroblasts	OKS	11R-fused CPPs			[44]
mNSCs	OKSMN	TiO2 Nanotube-mediated protein delivery		0.005%–0.01%	[49]
HDFs	OKSML with Vitamin C	MTDs	Partially reprogrammed	0.34%	[50]

cFBs: Cardiac fibroblasts; HAF: Human amniotic fluid; HDFs: Human dermal fibroblasts; HFFs: Human foreskin fibroblasts; HNFs: Human newborn fibroblast; MEFs: Mouse embryonic fibroblast; mNSCs: Mouse neural stem cells; NCCIT ECCs: NCCIT embryonic carcinoma cells; OKSM: Oct4, Klf4, Sox2, c-Myc; VPA: valproic acid; OS: Oct4, Sox2; PolyI:C: Polyinosinic:polycytidylic acid; TAT: Transactivator of transcription; OKS: Oct4, Klf4, Sox2; MTDs: macromolecule transduction domains.

## Abbreviations

CPP	Cell-penetrating peptide
PTD	Protein transduction domain
SCNT	Somatic cell nuclear transfer
MII	Metaphase II
iPSCs	Induced pluripotent stem cells
ESCs	Embryonic stem cells
SeV	Sendai virus
EC	Embryonic carcinoma
HIV-TAT	Human immunodeficiency virus trans-activator of transcription
FITC	Fluorescein isothiocyanate
HSCs	Hematopoietic stem cells
NLS	Nuclear localization signal
VPA	Valproic acid
TAT	Transactivator of transcription
HDFs	Human dermal fibroblasts
CE	Corneal endothelia
CendR	C-end rule
ADSCs	Adipose-derived stem cells
RPE	Reprogramming of pigmented epithelial
CPCs	Cardiac progenitor cells
MITT	Macromolecule intracellular transduction technology
MTDs	Macromolecule transduction domains
